# ICTV Virus Taxonomy Profile: Sphaerolipoviridae 2023

**DOI:** 10.1099/jgv.0.001830

**Published:** 2023-03-14

**Authors:** Tatiana A. Demina, Mike Dyall-Smith, Matti Jalasvuori, Shishen Du, Hanna M. Oksanen

**Affiliations:** 1 Department of Microbiology, Faculty of Agriculture and Forestry, and Helsinki Institute of Sustainability Science (HELSUS), University of Helsinki, Helsinki, Finland; 2 Computational Biology Group, Max Planck Institute of Biochemistry, Martinsried, Germany; 3 Veterinary Biosciences, Faculty of Veterinary and Agricultural Sciences, University of Melbourne, Parkville, Australia; 4 Department of Biological and Environmental Science, University of Jyväskylä, Jyväskylä, Finland; 5 Department of Microbiology College of Life Sciences, Wuhan University, Wuhan, Hubei, PR China; 6 Molecular and Integrative Biosciences Research Programme, Faculty of Biological and Environmental Sciences, University of Helsinki, Helsinki, Finland

**Keywords:** ICTV Report, *Sphaerolipoviridae*, Taxonomy

## Abstract

Members of the family *Sphaerolipoviridae* have non-enveloped tailless icosahedral virions with a protein-rich internal lipid membrane. The genome is a linear double-stranded DNA of about 30 kbp with inverted terminal repeats and terminal proteins. The capsid has a pseudo triangulation *T=*28 *dextro* symmetry and is built of two major capsid protein types. Spike complexes decorate fivefold vertices. Sphaerolipoviruses have a narrow host range and a lytic life cycle, infecting haloarchaea in the class Halobacteria (phylum Euryarchaeota). This is a summary of the International Committee on Taxonomy of Viruses (ICTV) Report on the family *Sphaerolipoviridae,* which is available at ictv.global/report/sphaerolipoviridae.

## Virion

**Fig. 1.**
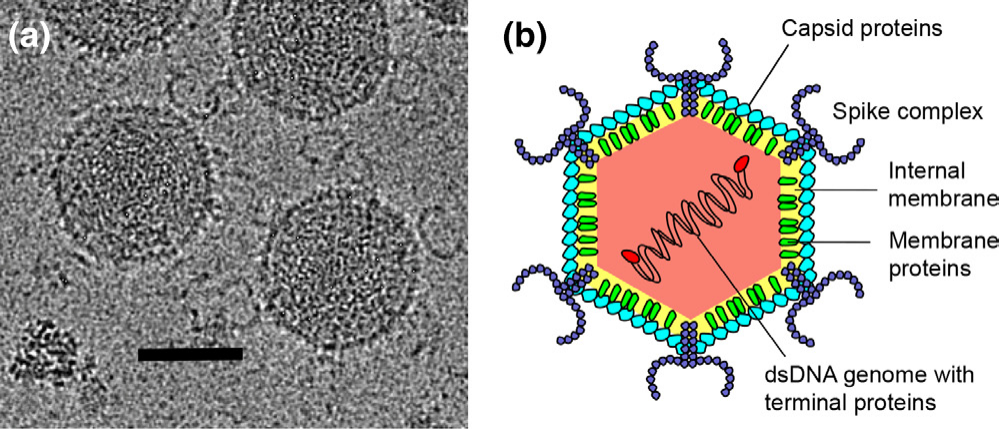
Morphology of Haloarcula californiae icosahedral virus 1 virion. (a) Cryo-electron micrograph of virions, scale bar: 50 nm, credit: Nicola Abrescia lab, CICbioGUNE, Spain. (b) Schematic model, modified from [6].

Sphaerolipoviruses have tailless icosahedral virions with an internal protein-rich membrane vesicle (Table 1; Fig. 1) [1,5]. The virion is typically about 80 nm in diameter with major and minor capsid proteins, internal membrane proteins and vertex complex proteins (Fig. 1). The capsid has a pseudo *T=*28 *dextro* triangulation number [4]. The two major capsid proteins (MCPs) VP4 and VP7 have a vertical single jelly-roll fold. The capsid lattice is built of pseudohexameric capsomers with either two or three towers (Fig. 1) made of VP4-VP4 homodimers and VP4-VP7 heterodimers [4]. The vertices are occupied by penton proteins forming the binding position for the spike complex. Vertex complexes are either horn-shaped or propeller-shaped [4]. MCPs, the major membrane protein, and the putative packaging ATPase are the most conserved structural proteins among sphaerolipoviruses [3,4]. The lipids of the internal membrane vesicle are selectively acquired from host-cell membranes. Membrane vesicles are rich in virus-specific proteins. Major phospholipid species are phosphatidylglycerol, phosphatidylglycerophosphate methyl ester and phosphatidylglycerosulfate [3].

**Table 1.** Characteristics of members of the family *Sphaerolipoviridae*

Example	Haloarcula californiae icosahedral virus 1 (KT809302), species *Alphasphaerolipovirus HCIV1,* genus *Alphasphaerolipovirus*
Virion	Non-enveloped, tailless icosahedral virion with an internal lipid membrane, diameter 80 nm, capsid is pseudo *T=*28 *dextro*, two types of major capsid protein, horn-shaped or propeller-shaped fivefold vertex spike complexes, membrane-associated proteins
Genome	Linear dsDNA, 28–31 kbp, with inverted terminal repeats and terminal proteins attached
Replication	Possibly protein-primed
Translation	Prokaryotic translation using viral mRNA and host ribosomes
Host range	Archaea, euryarchaeal *Haloarcula* and *Halorubrum* strains
Taxonomy	Realm *Varidnaviria*, kingdom *Helvetiavirae*, phylum *Dividoviricota*, class *Laserviricetes*, order *Halopanivirales*: one genus *Alphasphaerolipovirus* with several species

## Genome

**Fig. 2.**
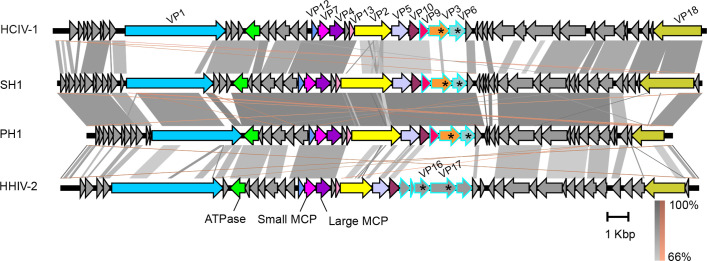
Genomes of sphaerolipoviruses. HCIV-1, Haloarcula californiae icosahedral virus 1 (31 314 bp, KT809302); SH1 virus, (30 889 bp, AY950802); PH1 virus, (28 072 bp, KC252997); HHIV-2, Haloarcula hispanica icosahedral virus 2 (30 578 bp, JN968479). ORFs/genes are shown as arrows. Homologous genes encoding structural proteins are highlighted with the same colours. Putative spike protein genes are marked with asterisks and ORFs encoding putative spike complex proteins are outlined with blue. Similarities between the genomes are shown as shadings of grey (direct) and brown (inverted). The figure was produced using Easyfig v.2.2.2 with the *E*-value threshold of 0.001.

Members of the family *Sphaerolipoviridae* have a linear double-stranded DNA genome of 28–31 kbp with a GC content of 67–68 % and inverted terminal repeats of about 300 bp with terminal proteins attached [2,3, 5]. Genomes contain about 50 predicted genes, arranged in a conserved synteny (Fig. 2) [2,3, 5]. The overall nucleotide identity between sphaerolipovirus genomes is 56–76 % [6].

## Replication

Replication is probably protein-primed [5], but the genome does not encode a canonical DNA polymerase. The genome of SH1 virus has genes organized in seven major transcripts, some of which overlap [5]. Six early transcripts encode structural genes, while one late transcript encodes proteins of unknown function. Spaherolipoviruses originate from hypersaline environments, and their host range is limited to a few haloarchaeal strains belonging to the genera *Haloarcula* and *Halorubrum* [6]. Sphaerolipoviruses bind to their hosts most probably by spike complexes at the virion vertices. Adsorption is relatively slow and the infection cycle is lytic, lasting 6–12 h [1,3]. Several putative proviral regions related to sphaerolipoviruses are found in the chromosomes of halophilic archaea [6].

## Taxonomy

Current taxonomy: ictv.global/taxonomy. The family *Sphaerolipoviridae* together with the families *Matsushitaviridae* (species *Hukuchivirus P2377* and *Hukuchivirus IN93*) and *Simuloviridae* (species *Yingchengvirus SNJ1, Yingchengvirus NVIV1* and *Yingchengvirus HJIV1*) are assigned to the order *Halopanivirales*.

## Resources

Full ICTV Report on the family *Sphaerolipoviridae*: ictv.global/report/sphaerolipoviridae.

## Abbreviation

MCP: major capsid protein
